# Edentulousness among Patients Visiting a Dental Unit of a Tertiary Care Centre: A Descriptive Cross-sectional Study

**DOI:** 10.31729/jnma.8017

**Published:** 2023-02-28

**Authors:** Lochana Acharya, Abhishek Gupta, Prabhat Shrestha, Sijan Poudyal, Sabina Poudel

**Affiliations:** 1 Department of Prosthodontics, KIST Medical College and Teaching Hospital, Gwarko, Lalitpur, Nepal; 2 Department of Oral Medicine and Radiology, Chitwan Medical College and Teaching Hospital, Bharatpur, Chitwan, Nepal; 3 Department of Prosthodontics, KIST Medical College and Teaching Hospital, Gwarko, Lalitpur, Nepal; 4 Department of Community Dentistry, People's Dental College and Hospital, Kathmandu, Nepal; 5 Aadhya Dental and Implant Centre, Kathmandu, Nepal

**Keywords:** *dental health service*, *edentulous mouth*, *Nepal*, *prevalence*

## Abstract

**Introduction:**

Edentulousness (partial or complete) is a sequel of tooth loss and is an indicator of the oral health status of a population. Edentulousness has a series of deleterious consequences for oral and genera! health. The aim of this study was to find out the prevalence of edentulousness among patients visiting the dental unit of a tertiary care centre.

**Methods:**

A descriptive cross-sectional study was carried out based on hospital records of patients visiting the Department of Oral Medicine and Prosthodontics of a tertiary care centre from 1 January 2019 to 30 December 2019 to see the prevalence of edentulousness. Ethical approval was obtained from the Institutional Review Committee (Reference number: 077/ 078 /40). A convenience sampling method was used. Point estimate and 95% Confidence Interval were calculated.

**Results:**

Among 4697 patients, edentulousness was found in 403 (8.58%) (7.78-9.38, 95% Confidence Interval). Partial edentulous were 263 (65.30%) and complete edentulous were 140 (34.70%). Of the total partial edentulous patient, Kennedy's class III found in 200 (76.05%) was the most common pattern followed by Kennedy's class I in 32 (12.17%), class II in 21 (7.98%) and class IV in 10 (3.80%) patients respectively.

**Conclusions:**

The prevalence of edentulousness was similar to other studies done in similar settings. Since edentulousness is a preventable problem, it should be addressed with high priority.

## INTRODUCTION

Absence of teeth known as Edentulism is a common oral health issue worldwide that accounts for approximately 1/3^rd^ of the total oral disorders.^1,2^ It is described as the "final marker of disease burden for oral health".^3^ The prevalence of edentulism has declined significantly in many countries over the recent decades,^4,5^ it still remains a major disease worldwide, especially among older adults.^6^

According to the WHO definition, a person who is edentulous is deemed to be handicapped.^7^ So edentulousness being a preventable oral disability, it should be identified and awareness of its management should be done in order to bring a positive change in the health of individuals. In Nepal, edentulism is considered a consequence of old age and the data on its prevalence is scarce.

The aim of the study was to estimate the prevalence of edentulousness among the patients visiting the Dental unit of the tertiary care centre.

## METHODS

A descriptive cross-sectional study was conducted in the Department of Oral Medicine and Radiology and Prosthodontics of the Dental unit at KIST Medical College and Teaching Hospital for a period of 1 year from 1 January 2019 to 30 December 2019 using patient records. Ethical approval was obtained from the Institutional Review Committee (Reference number: 077/ 078 /40) from the same institution. The inclusion criteria for the study were patients visiting the dental unit during the study period. The exclusion criteria were the record with incomplete demographic or clinical data, patients aged below 20 years and follow-up cases. A convenience sampling method was used. The sample size was calculated by using the following formula:


$$\begin{array}{ll} \text{n} & ={\text{Z}}^{2}\times \frac{\text{p}\times \text{q}}{{\text{e}}^{\text{2}}}\\ & =1.96^{2}\times \frac{0.50\times 0.50}{0.03^{2}}\\ & =1068 \end{array}$$

Where,

n = minimum required sample size
Z = 1.96 at 95% Confidence Interval (CI)
p = prevalence taken as 50% for maximum sample size calculation
q = 1-p
e = margin of error, 3%

The minimum required sample size was 1068. As the convenience sampling method was used, the sample was quadrupled and 4269 was assumed. However, a total of 4697 patients were included in the study.

Edentulism is the state of being without natural teeth and is classified as complete and partial.^1^ The data for the study were collected from the patient record on predesigned Proforma.

Data were analyzed by using IBM SPSS Statistics 20.0. Point estimate and 95% CI were calculated.

## RESULTS

**Figure 1:**
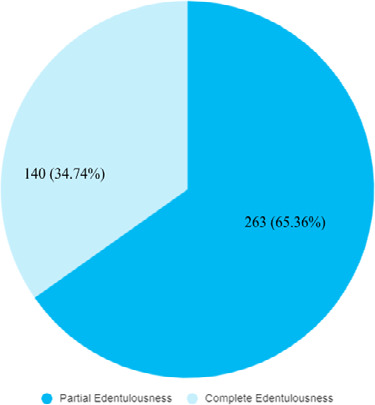
Distribution of partial and complete edentulousness (n= 403).

Among 4697 patients, edentulousnesswas found in 403 (8.58%) (7.78-9.38, 95% CI). Among them, 263 (65.36%) were partial edentulous and 140 (34.74%) were complete edentulous (Figure 1).

Out of 263 partial edentulous patients, Kennedy's Class III was the most common pattern seen in 200 (76.05%), followed by Kennedy's class I in 32 (12.17%) (Table 1).

**Table 1:** Distribution of partial edentulousness according to Kennedy’s classification (n= 403)

Kennedy's classification	n (%)
Class I	32 (12.17)
Class II	21 (7.98)
Class III	200 (76.05)
Class IV	10 (3.80)

## DISCUSSION

Among 4697 patients, edentulousness was found in 8.58% which is similar to the previous study conducted in Nepalese population (8.7%).^8^ However, the prevalence of edentulism varies from one country to another country and from one region to another region,^9^ and comparing between national samples is challenging because of the impact of several factors like lifestyle, socio-economic circumstances, education, oral health knowledge and beliefs, and attitudes toward dental care.^10^

Out of 403 total edentulous patients, 65.36% were partially edentulous which is almost similar to the finding of the study conducted in the Saudi Arabia population 69% and the Nepalese population 64%.^11,12^ In this study 140 (34.74%) were completely edentulous which seems to be higher. An extensive survey study that was conducted to assess complete edentulism among older adults, 50 years and above, in several countries, namely India, Ghana, China, Mexico, Russia, and South Africa showed that Mexico had the highest prevalence at 21.7% with Russia coming in second at 18% and India third at 16.3%. China and South Africa were similar at 9% and 8.5%, respectively. The least prevalence rate for edentulism was seen in Ghana (3%).^13^ The variation in finding may be attributed to several factors like age, socioeconomic status, geographical variation and oral health awareness of the population.

In this study, Kennedys class III is the most prevalent pattern of partial edentulousness which is similar to the previous study conducted in the Nepalese population,^12,14-16^ and similar results were obtained in a previous study conducted in the Iraqi population.^17,18^ This could be attributed to the fact that the first molar is the first permanent tooth to erupt into the oral cavity and has a higher chance of being affected by caries and a greater likelihood of the tooth being extracted.^19^ However, few studies have shown Class I as the most prevalent type of partial edentulism in their study. Kennedys class IV is the least common type of edentulousness in our study which is similar to the previous study conducted on the Kashmir population.^18,19^

This study was conducted in a single centre and patients enrolled using a convenience sampling method so the findings might not be generalizable to a larger population. Further research with a multicenter study could be done for the estimation of the prevalence.

## CONCLUSIONS

The prevalence of edentulousness is similar to the finding of other studies done in similar settings. Since it is a preventable disease, it should be identified and managed early to increase the quality of life. It would be valuable information to the National Oral Health Planners for laying out strategies to develop dental health care management on edentulousness in the country.

## References

[1] The Glossary of Prosthodontic Terms: Ninth Edition. (2017). J Prosthet Dent..

[2] Global Burden of Disease Study 2013 Collaborators. (2015). Global, regional, and national incidence, prevalence, and years lived with disability for 301 acute and chronic diseases and injuries in 188 countries, 1990-2013: a systematic analysis for the Global Burden of Disease Study 2013.. Lancet..

[3] Cunha-Cruz J, Hujoel PP, Nadanovsky P (2007). Secular trends in socio-economic disparities in edentulism: USA, 1972-2001.. J Dent Res..

[4] Madhankumar S, Mohamed K, Natarajan S, Kumar VA, Athiban I, Padmanabhan TV, Madhankumar S (2015). Prevalence of partial edentulousness among the patients reporting to the department of prosthodontics Sri Ramachandra University Chennai, India: an epidemiological study.. J Pharm Bioallied Sci..

[5] Vadavadagi SV, Srinivasa H, Goutham GB, Hajira N, Lahari Vadavadagi SV, Srinivasa H (2015). Partial edentulism and its association with socio-Demographic variables among subjects attending dental teaching institutions, India.. J Int Oral Health..

[6] Douglass CW, Shih A, Ostry L (2002). Will there be a need for complete dentures in the United States in 2020?. J Prosthet Dent..

[7] Marcus PA, Joshi A, Jones JA, Morgano SM (1996). Complete edentulism and denture use for elders in New England.. J Prosthet Dent..

[8] Basnyat KC, Sapkota B, Shrestha S (2014). Epidemiological survey on edentulousness in elderly Nepalese Population.. Kathmandu Univ Med J (KUMJ)..

[9] Millar WJ, Locker D (2005). Edentulism and denture use.. Health Rep..

[10] Muller F, Naharro M, Carlsson GE (2007). What are the prevalence and incidence of tooth loss in the adult and elderly population in Europe?. Clin Oral Implants Res..

[11] Almusallam SM, AlRafee MA (2020). The prevalence of partial edentulism and complete edentulism among adults and above population of Riyadh city in Saudi Arabia.. J Family Med Prim Care..

[12] Pradhan D, Shrestha L, Dixit S, Shrestha A (2017). Prevalence of type of partial edentulousness among the population of Bhotenamlang, Sindhupalchowk, Nepal: an observational study.. International Journal of Science and Research (IJSR)..

[13] Peltzer K, Hewlett S, Yawson AE, Moynihan P, Preet R, Wu F (2014). Prevalence of loss of all teeth (edentulism) and associated factors in older adults in China, Ghana, India, Mexico, Russia and South Africa.. Int J Environ Res Public Health..

[14] Sapkota B, Adhikari B, Upadhaya C (2013). A study of assessment of partial edentulous patients based on Kennedy's classification at Dhulikhel Hospital Kathmandu University Hospital.. Kathmandu Univ Med J (KUMJ)..

[15] Rana SB, Acharya B, Bhochhibhoya A, Sharma R, Acharya J, Mainali A (2018). Patterns of partial edentulism based on Kennedy's classification among patients reporting to Nepal Medical College and Teaching Hospital.. Journal of Kathmandu Medical College..

[16] Manandhar P, Ranjit R, Tuladhar SL, Bhandari A (2021). Prevalence of partial edentulism among the patients visiting a tertiary health care center in the Western region, Nepal.. Journal of Gandaki Medical college Nepal..

[17] Hatim NA, Muhammed SA, Hasan NH (2003). Psychosocial profile of patient with missing teeth and refuses treatment.. Al-Rafidain Dental Journal..

[18] Fayad MI, Baig MN, Alrawaili AM (2016). Prevalence and pattern of partial edentulism among dental patients attending College of Dentistry, Aljouf University, Saudi Arabia.. J Int Soc Prev Community Dent..

[19] Lone MA, Shah SA, Mir S (2019). Pattern of partial edentulism based on Kennedys classification among dental patients in Kashmir: retrospective study.. International Journal of Applied Dental Sciences..

[20] Araby YA, Almutairy AS, Alotaibi FM (2017). Pattern of partial edentulism in correlation to age and gender among a selected Saudi population.. International Journal of Dental Sciences and Research..

[21] Abdel-Rahman HK, Tahir CD, Saleh M (2013). Incidence of partial edentulism and its relation with age and gender.. Zanco J Med Sci..

